# Towards improving tsetse fly paratransgenesis: stable colonization of *Glossina morsitans morsitans* with genetically modified *Sodalis*

**DOI:** 10.1186/s12866-018-1282-9

**Published:** 2018-11-23

**Authors:** Linda De Vooght, Severien Van Keer, Jan Van Den Abbeele

**Affiliations:** Department of Biomedical Sciences, Unit of Veterinary Protozoology, Institute of Tropical Medicine Antwerp, Antwerp, Belgium

**Keywords:** Paratransgenesis, *Glossina*, *Sodalis glossinidius*, Colonization, Transmission, GFP

## Abstract

**Background:**

Tsetse flies (*Glossina* sp.) refractory to trypanosome infection are currently being explored as potential tools to contribute in the control of human and animal African trypanosomiasis. One approach to disrupt trypanosome transmission by the tsetse fly vector involves the use of paratransgenesis, a technique that aims to reduce vector competence of disease vectors via genetic modification of their microbiota. An important prerequisite for developing paratransgenic tsetse flies is the stable repopulation of tsetse flies and their progeny with its genetically modified *Sodalis* symbiont without interfering with host fitness.

**Results:**

In this study, we assessed by qPCR analysis the ability of a chromosomally GFP-tagged *Sodalis* (rec*Sodalis*) strain to efficiently colonize various tsetse tissues and its transmission to the next generation of offspring using different introduction approaches. When introduced in the adult stage of the fly via thoracic microinjection, rec*Sodalis* is maintained at high densities for at least 21 days. However, no vertical transmission to the offspring was observed. Oral administration of rec*Sodalis* did not lead to the colonization of either adult flies or their offspring. Finally, introduction of rec*Sodalis* via microinjection of third-instar larvae resulted in stably colonized adult tsetse flies. Moreover, the subsequent generations of offspring were also efficiently colonized with rec*Sodalis*. We show that proper colonization of the female reproductive tissues by rec*Sodalis* is an important determinant for vertical transmission.

**Conclusions:**

Intralarval microinjection of rec*Sodalis* proves to be essential to achieve optimal colonization of flies with genetically modified *Sodalis* and its subsequent dissemination into the following generations of progeny. This study provides the proof-of-concept that *Sodalis* can be used to drive expression of exogenous transgenes in *Glossina morsitans morsitans* colonies representing a valuable contribution to the development of a paratransgenic tsetse fly based control strategy.

**Electronic supplementary material:**

The online version of this article (10.1186/s12866-018-1282-9) contains supplementary material, which is available to authorized users.

## Background

As vector-borne diseases continue to present significant threats to human, animal and plant health, there is a perpetual need to improve existing and develop new control strategies. Vector control has always been and still remains a key component of any integrated program to control vector-borne diseases. In the light of increasing insecticide resistance combined with the growing awareness of the negative environmental and ecological consequences caused by the widespread use of insecticides, genetic control of the insect vector provides a valuable addition to the armory of tools already available.

Tsetse flies (*Glossina* sp.) are medically and agriculturally important vectors that transmit *Trypanosoma* spp. parasites responsible for human sleeping sickness and animal African trypanosomiasis (AAT). Today, prevention and control programs are mainly based on elimination of the parasite reservoir and vector-oriented control since there are no prophylactic drugs or vaccines available and the few available treatments present serious side effects [1, 2]. The generation of a trypanosome-resistant tsetse fly that is incapable of transmitting the trypanosome parasite would be extremely valuable in integrated control programs against African Trypanosomiasis by complementing the sterile insect technique (SIT) that has proven effective in eradicating tsetse in isolated pockets [3]. At present, SIT relies on the massive release of sterile male tsetse flies accompanied by a temporary increase in the number of potential vectors for trypanosomes, especially during the first few years of the control campaign. As such, the use of tsetse flies with a refractory phenotype would render this approach less controversial especially when applied in areas where human sleeping sickness is also occurring beside AAT.

Since tsetse flies are not amenable to germ-line transformation due to their viviparous reproductive biology, a paratransgenic approach using the tsetse fly secondary symbiont *Sodalis glossinidius* as a delivery system for anti-trypanosomal components is currently of considerable interest to achieve a refractory phenotype. *Sodalis glossinidius* is a maternally inherited gram-negative bacterial endosymbiont of the tsetse fly that can be found both inter- and intracellularly in the tsetse fly midgut, muscle, fat body, milk glands, and salivary glands [4]. A crucial step in developing paratransgenic tsetse is the stable colonization of flies and their progeny with recombinant *Sodalis* strains expressing trypanosome-interfering proteins in insect tissues where trypanosome parasites reside. The current method to introduce recombinant *Sodalis* into the tsetse fly relies on thoracic microinjection into the haemolymph [5].

Recently, we developed a plasmid-based expression system allowing *Sodalis* to constitutively express and release functional trypanosome-binding nanobodies (Nbs) targeting the *Trypanosoma brucei* VSG, in an in vitro culture system as well as in vivo in different tissues of the tsetse fly following introduction via thoracic microinjection [6, 7]. However, our experiments showed that when introducing the plasmid-bearing *Sodalis* into adult tsetse flies by intrathoracic injection, transmission to the progeny is limited, hampering the establishment of a paratransgenic tsetse fly colony. This inefficient transfer upon injection in the adult female fly could be attributed to plasmid loss by the recombinant *Sodalis* and/or its inability to efficiently colonize the female milk glands which is a prerequisite for recombinant *Sodalis* transmission to the intra-uterine larvae through the maternal milk secretion. To overcome the need for continuous selection pressure inherent to plasmid-based gene expression, we recently developed a Tn7-based transposition system for the stable integration of transgenes into a transcriptional highly active region of the *Sodalis* genome [8].

In this study, we investigated alternative approaches to stably colonize tsetse flies and their subsequent generations with recombinant *Sodalis* using a genetically engineered *Sodalis* strain constitutively expressing green fluorescent protein (GFP) for the proof-of-concept. First we assessed the suitability of *Sodalis* native and non-native promoters for the chromosomal expression of GFP using flow cytometry. Next we evaluated the ability of GFP-tagged *Sodalis* (rec*Sodalis*) to efficiently colonize various tsetse tissues after being introduced in the fly via two different approaches; i) introduction in wild type (WT) *Sodalis* cleared adult female flies either via thoracic microinjection or blood meal supplementation and ii) microinjection of third-instar larvae. Besides colonization of the F_0_ generation, vertical transmission to and colonization of the progeny by the rec*Sodalis* strain along multiple generations was investigated. We demonstrate that intralarval microinjection of rec*Sodalis* proves to be essential to achieve maternal transfer and subsequent establishment of a genetically modified *Sodalis* strain into the following generations of progeny.

## Results

### Chromosomal expression of a reporter gene controlled by a native or a heterologous constitutive promoter

**Fig. 1:**
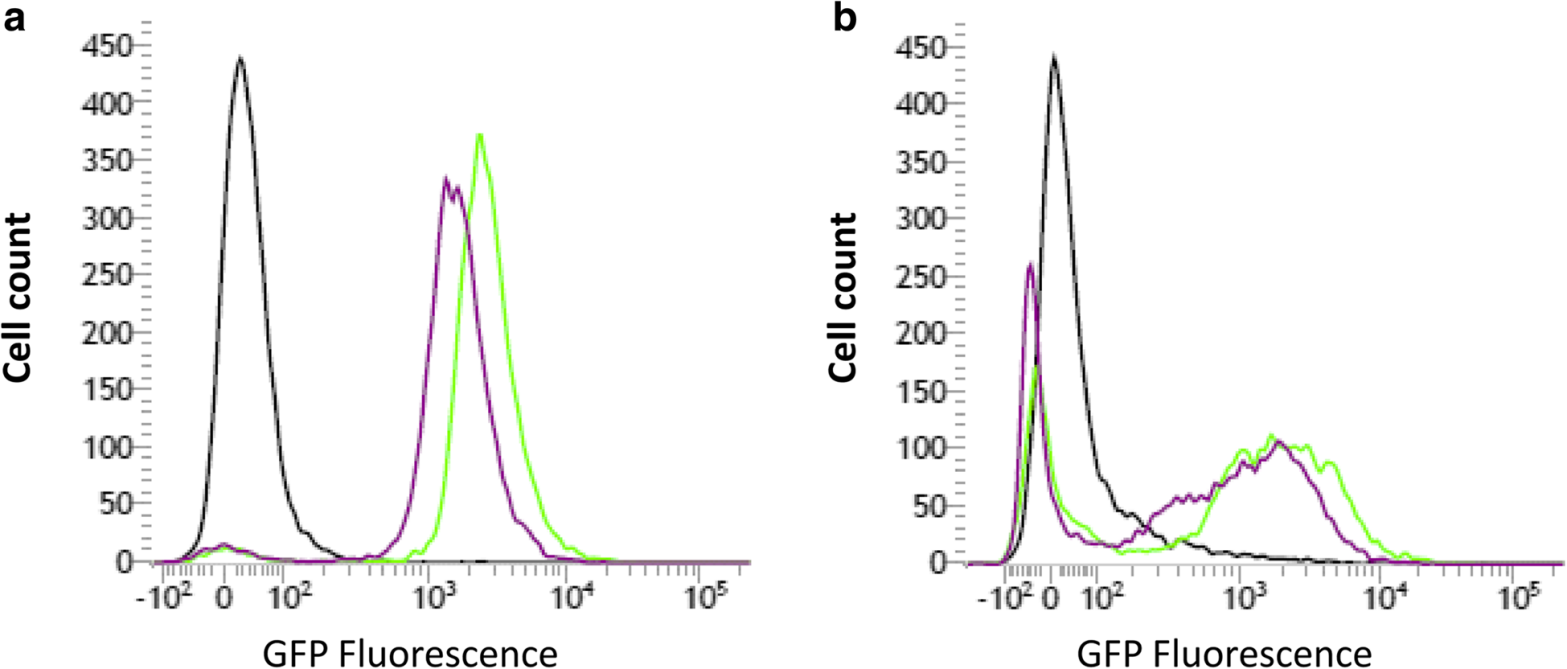
**a** FACS plot overlay showing in vitro green fluorescent protein (GFP) expression of WT *Sodalis* cells (black histogram), chromosomally GFP-tagged *Sodalis* cells harboring the *E. coli* lacZ promotor (green histogram) and chromosomally GFP-tagged *Sodalis* cells harboring the *Sodalis* GroEl promotor (purple histogram); **b** GFP expression of *Sodalis* in WT flies (black histogram) and flies injected with chromosomally GFP-tagged *Sodalis* cells harboring the *E. coli* lacZ promotor (green histogram) and the *Sodalis* GroEl promotor (purple histogram). The low intensity peaks result from the native WT *Sodalis* population in injected flies

We assessed the capability of two different promotors i.e., the *E. coli* lac Z and the native *Sodalis glossinidius groEL* promoter, to chromosomally express GFP in *Sodalis* in in vitro culture conditions as well as in vivo. Since *Sodalis* is devoid of a *lac* repressor, the presence of the *E. coli lac* promoter results in a constitutive expression of the transgene. The universal heat shock chaperonin GroEL is constitutively expressed at high levels in *Sodalis* by its efficient promoter. Both the *E. coli* lacZ as well as the *Sodalis* GroEL promotor proved to be functional for the expression of active GFP in in vitro conditions as well as in vivo in the haemolymph. Flow cytometry analysis showed that chromosomal GFP expression under control of the lacZ promotor resulted in higher expression levels in vitro as well as in vivo compared to the GroEL promotor (Fig. 1). Since promoters that drive the strong expression of effector genes in transgenic insects are likely to result in more abundant transcription and increased effectiveness, the lacZ promotor was used for all future experiments involving the expression of effector genes in *Sodalis*.

### Recombinant symbiont introduction into adult tsetse flies

From our previous studies we know that transient treatment of *G. morsitans morsitans* flies with streptozotocin reduces the WT *Sodalis* population which is crucial for allowing recombinant *Sodalis* to proliferate inside its host without affecting the obligatory *Wigglesworthia* symbiont population [7].

Prior to the introduction of rec*Sodalis*, female flies received 3 streptozotocin (20 μg/ml) supplemented blood meals reducing the WT *Sodalis* population by 98, 97 and 93% in abdomen, thorax and female reproductive organs respectively, compared to flies fed on normal blood (Additional file 1). Next, we analyzed the ability of rec*Sodalis* to colonize different tissues of streptozotocin-treated female tsetse flies and its transmission to the progeny after introduction via either thoracic microinjection, blood meal supplementation or a combination of the two. This analysis was done by qPCR based estimation of the amount of rec*Sodalis* CFU present in abdomen, thorax and reproductive organs over a 21 day period.

**Fig. 2:**
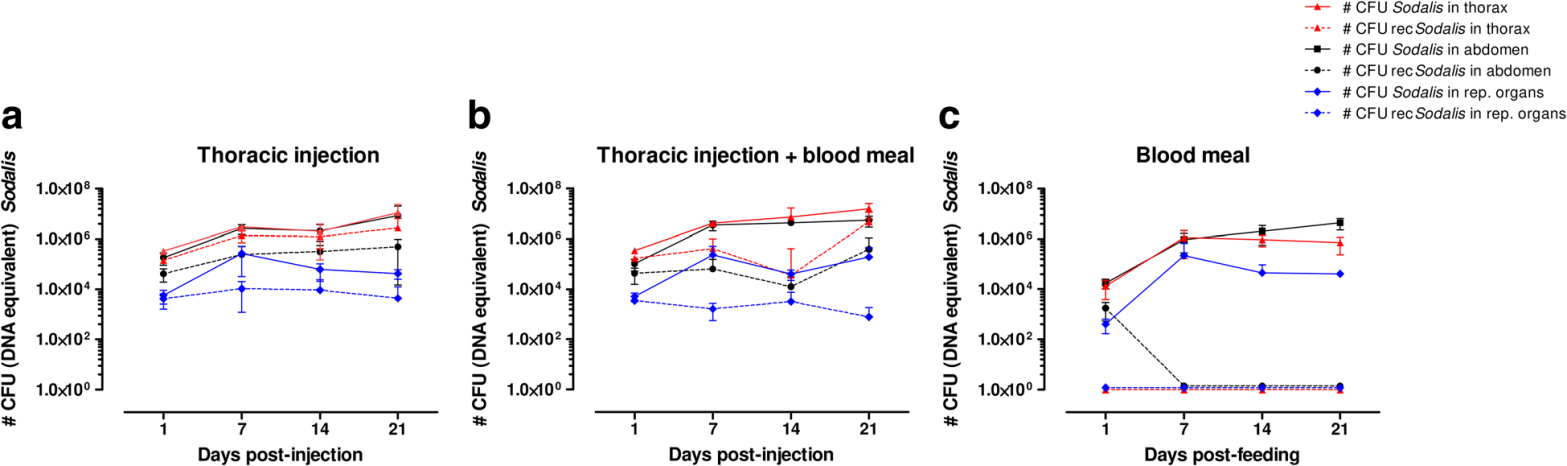
Evaluating host colonization after introduction of rec*Sodalis* via **a** thoracic injection, **b** thoracic injection combined with blood meal supplementation and **c** blood meal supplementation only. Graphs depict the number of rec*Sodalis* CFU (DNA equivalent) present in abdomen (dashed black line), thorax (dashed red line) and reproductive organs (dashed blue line) versus the total number of *Sodalis* CFU (DNA equivalent) (WT + recombinant *Sodalis*) present in abdomen (solid black line), thorax (solid red line) and reproductive organs (solid blue line). Data points represent the mean rec*Sodalis* and total *Sodalis* CFU (± SD) present in the different tissues of individual flies (*n* = 5) at the time of sampling. The number of CFU is represented in log scale on the y-axis

Rec*Sodalis* was able to proliferate in abdomen and thorax tissues of flies injected intrathoracically with 5 × 10^6^ rec*Sodalis* CFU and remained present at high densities throughout the course of the 21-day observation period (Fig. 2a). A similar colonization pattern was observed in flies receiving both a thoracic injection and a rec*Sodalis* supplemented blood meal with rec*Sodalis* densities persisting at 10^5^ and 10^6^ CFU (DNA equivalent) in abdomen and thorax tissues respectively throughout the 21-day observation period (Fig. 2b). For both conditions, rec*Sodalis* could be detected in the female reproductive organs, however colonization was limited with less than 10% of the total *Sodalis* population proving to be recombinant (Fig. 2a and b). Rec*Sodalis* offered to flies via only blood meal supplementation was not able to maintain itself in the fly: 24 h post-feeding rec*Sodalis* could not be detected in either abdomen, thorax or reproductive tissues (Fig. 2c).

The streptozotocin treatment had a negative effect upon tsetse fecundity; in all experimental groups an arrest in the reproductive cycle of the females was seen for ~ 21 days, after which only a proportion of the experimental female flies that received rec*Sodalis* via thoracic injection combined with blood meal supplementation and blood meal supplementation only started to produce offspring again (respectively 50% and 46% of the female flies). qPCR showed that streptozotocin had no effect on *Wigglesworthia*, the primary endosymbiont of tsetse known to be important for fertility. Upon dissection of the reproductive organs of sterile females we did observe atrophy of the ovaries suggesting that the observed reduction in fecundity resulted from a more direct effect on the host rather than on its primary symbiont.

**Fig. 3:**
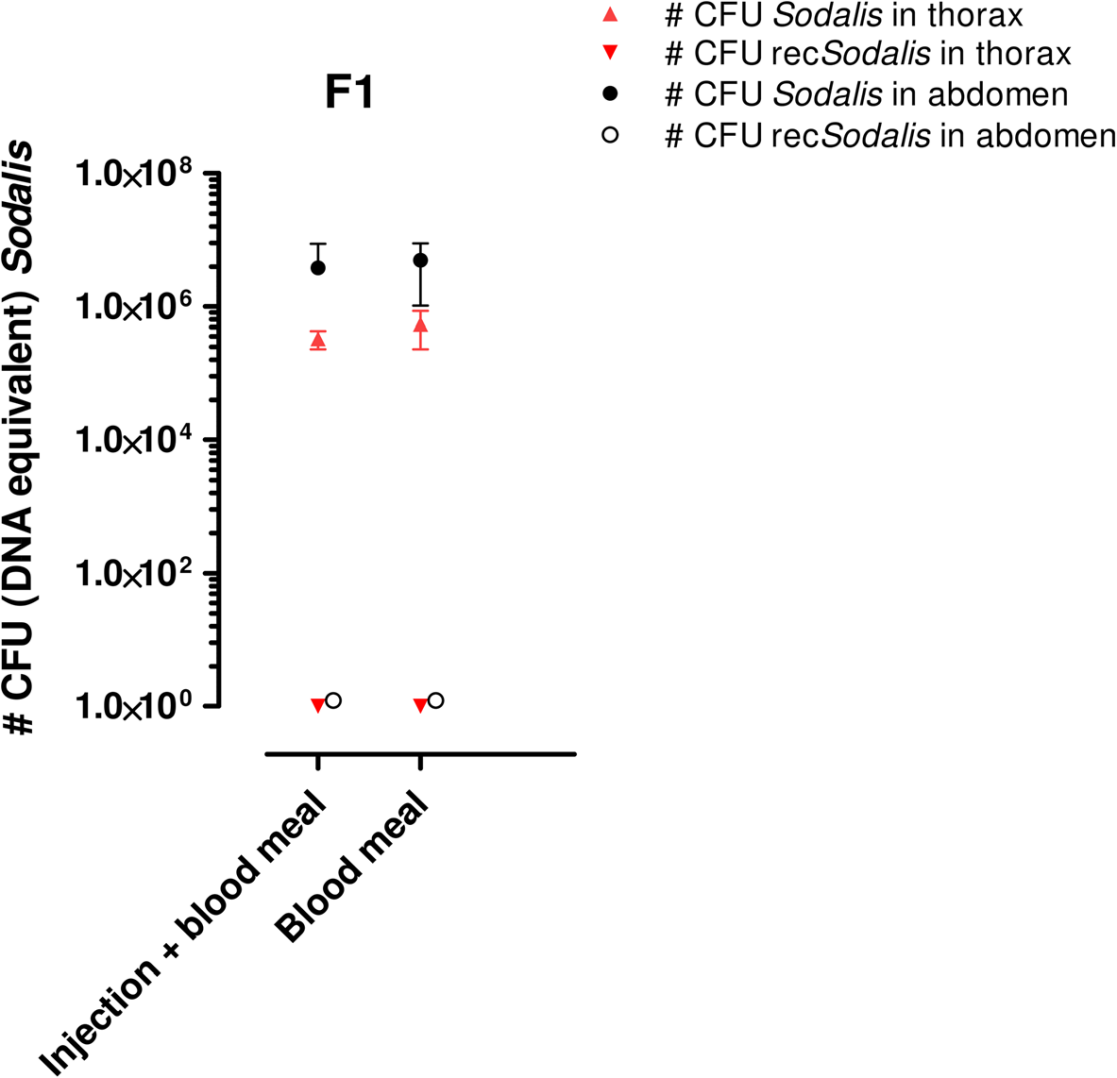
Evaluating transmission to the offspring after introduction in adult stages. rec*Sodalis* could not be detected in the abdomen or thorax of the first generation male progeny (F_1_) of female flies treated with GFP-tagged *Sodalis* via a combination of injection and feeding or feeding alone. Data points represent the mean total *Sodalis* CFU (± SD) present in the different tissues of individual flies (*n* = 5) at the time of sampling. The number of CFU is represented in log scale on the y-axis

Finally, transmission dynamics of the recombinant bacteria to the progeny were also evaluated by qPCR; no vertical transmission was observed as rec*Sodalis* could not be detected in the F1 generation of any of the mother flies that received rec*Sodalis* via intrathoracic injection or feeding (Fig. 3).

### Recombinant symbiont introduction into third-instar larvae

**Fig. 4:**
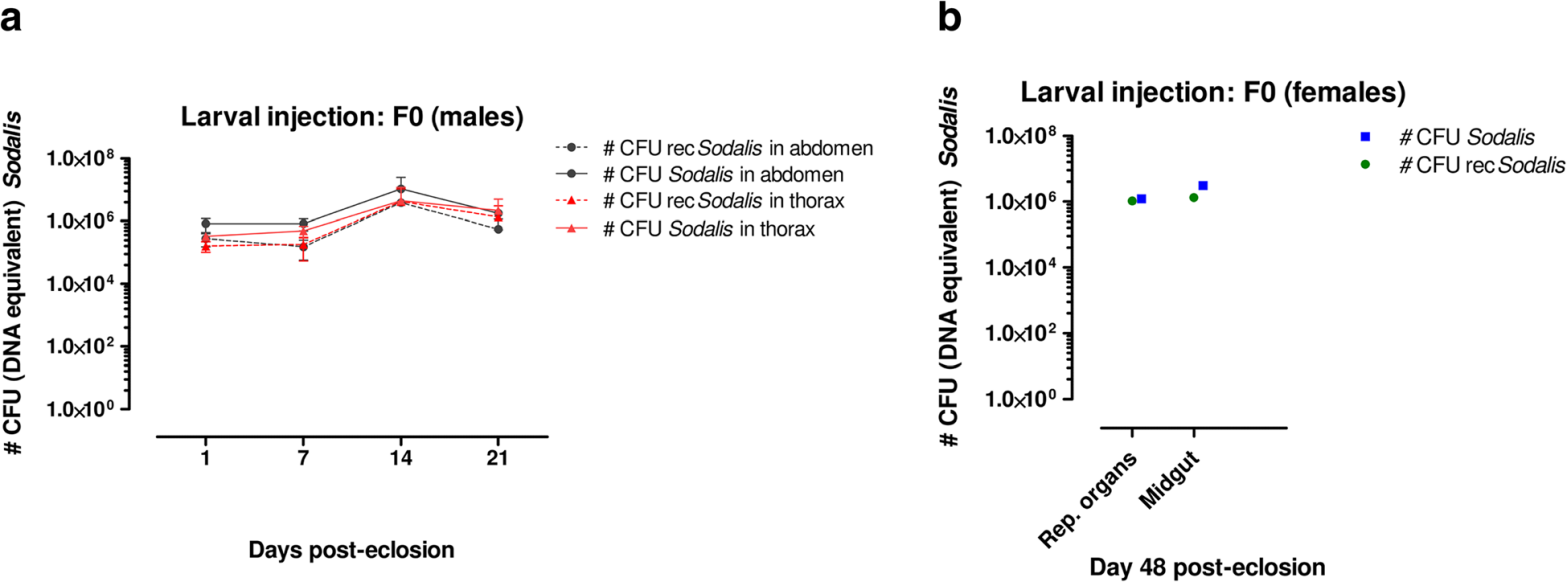
Evaluating host colonization after introduction of rec*Sodalis* via larval injection. **a** the number of rec*Sodalis* CFU (DNA equivalent) present in abdomen (dashed black line) and thorax (dashed red line) tissues of male flies emerged from larvae injected with 1 × 10^6^ recombinant CFU versus the total number of *Sodalis* CFU DNA equivalent (WT + recombinant *Sodalis*) present in abdomen (solid black line) and thorax tissues (solid red line). **b** the number of rec*Sodalis* CFU (DNA equivalent) present in female reproductive tissues and midguts (green) versus the total number of *Sodalis* CFU DNA equivalent (WT + recombinant *Sodalis*) (blue). Data points represent the mean rec*Sodalis* and total *Sodalis* CFU (± SD) present in the different tissues of individual flies (*n* = 5) at the time of sampling. The number of CFU is represented in log scale on the y-axis

Third-instar larvae, collected immediately after larviposition were injected with 10^6^ CFU of rec*Sodalis*. Pupated larvae were allowed to hatch and the recombinant as well as the total *Sodalis* densities present in abdomen and thorax tissues from the emerged male adult flies (F_0_) were measured using qPCR. Emerged female flies were mainly kept for mating to assess the vertical transmission of rec*Sodalis* to the offspring, therefore colonization of rec*Sodalis* in female reproductive organs and midgut was only assessed on day 48 post-eclosion. The abdomen and thorax from male flies that emerged from larvae (F_0_) injected with 1 × 10^6^ CFU (Fig. 4a) were efficiently colonized by rec*Sodalis* from day 1 post-emergence with the population growing to peak densities of around 4 × 10^6^ CFU (DNA equivalent) on day 14 in both abdomen and thorax tissues. The reproductive organs of female flies emerged from injected larva were efficiently colonized with 87% of the total *Sodalis* population being recombinant, reaching a 10^3^-fold higher density compared to the thoracic introduction method (Fig. 4b).

**Fig. 5:**
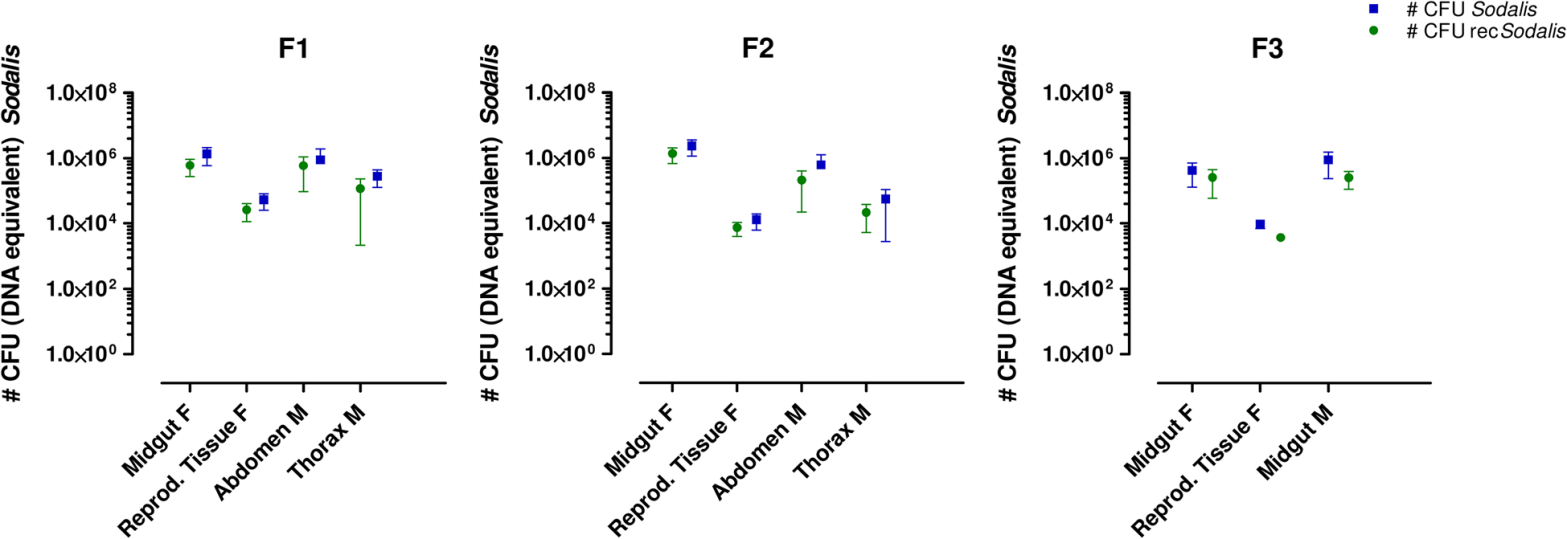
Evaluating transmission to the offspring after introduction in larval stages. rec*Sodalis* was found to be present in different tissues of 1st, 2nd and 3rd generation of male (M) and female (F) offspring resulting from female flies microinjected with 1 × 10^6^ CFU rec*Sodalis* at the larval stage. Graphs depict the number of rec*Sodalis* CFU (DNA equivalent) present in female and males tissues (green) versus the total number of *Sodalis* CFU (DNA equivalent) (WT + recombinant *Sodalis*) (blue). Data points represent the mean rec*Sodalis* and total *Sodalis* CFU (± SD) present in the different tissues of individual flies (*n* = 5) at the time of sampling; 4 days post-eclosion. The number of CFU is represented in log scale on the y-axis

Furthermore, rec*Sodalis* was transmitted to both male and female offspring resulting from female flies microinjected at the larval stage (Fig. 5). We detected rec*Sodalis* to be present in densities comparable to natural *Sodalis* levels present in WT flies in all tissues including abdomen, thorax, female reproductive tissues and midgut. We continued to breed from the offspring until the F3 generation (around 150 days after the initial F_0_ larvae injection) and we observed that all flies remained highly positive for rec*Sodalis.*

## Discussion

Despite the interest in *Sodalis* as an in vivo drug delivery vehicle to control trypanosome parasite development in the tsetse fly, significant hurdles still need to be overcome towards achieving this goal. The stable colonization of adult flies and their progeny with the introduced genetically modified *Sodalis* strain during multiple generations is an important prerequisite for *Sodalis* to be used as a paratransgenic platform. Therefore, in this study we aimed to optimize the existing *Sodalis* re-introduction methods and evaluate the colonization efficiencies of the re-introduced bacteria in the tsetse fly along subsequent generations. Using qPCR analysis we assessed the ability of a chromosomally GFP-tagged *Sodalis* (rec*Sodalis*) strain to efficiently colonize various tsetse tissues after being introduced in the tsetse fly via two different approaches; i) introduction in WT *Sodalis* cleared adult female flies either via thoracic microinjection or blood meal supplementation and ii) introduction in third-instar larvae. Besides colonization of the F_0_ generation, vertical transmission to and colonization of the progeny by the rec*Sodalis* strain was also investigated.

When introduced via thoracic microinjection in the haemolymph of adult flies cleared of WT *Sodalis*, rec*Sodalis* was able to establish and proliferate in both abdomen and thorax of injected flies. However, the rec*Sodalis* population predominantly remained present locally at the site of injection i.e. in the haemolymph. Indeed, the lower number of rec*Sodalis* observed in female reproductive organs indicates limited dissemination of rec*Sodalis* to these tissues via the haemolymphatic circulation of the fly. We next investigated an alternative, oral route of recombinant symbiont introduction in adult tsetse. Maltz et al. [9] were able to efficiently colonize tsetse fly gut tissue with mutant *Sodalis* via a complement inactivated blood meal, supplemented with 500 CFU/ml *Sodalis*. However, all attempts to colonize tsetse fly tissues by this oral administration route were not successful in our study.

Both the thoracic microinjection and oral introduction routes of rec*Sodalis* in adult females did not result in the vertical transmission of the bacterium to the offspring. From the above described experiments where rec*Sodalis* is introduced in adult female flies we can conclude that rec*Sodalis* predominantly remains present at the site of injection with little dissemination to the reproductive organs and no vertical transfer to the offspring. This suggests that the ability of the rec*Sodalis* bacterium to reach and colonize the reproductive organs (milk glands, ovarial tissue) is compromised in the adult fly stage. Unlike many other insects, tsetse flies display a viviparous mode of reproduction where the female fly gives birth to a full grown (third-instar) larva that has been nourished within the female uterus by maternal milk gland secretions containing essential nutrients for larval development as well as the fly’s symbionts *Wigglesworthia* and *Sodalis*. In the milk gland, *Sodalis* resides both extracellularly in the lumen as intracellularly within the milk gland secretory cells [10]. Cell invasion has been shown to play an important factor for *Sodalis* to be vertically transmitted to the offspring. In a mutant line of *Sodalis*, lacking a crucial type III secretion system invasion gene (*invC*), establishment of *Sodalis* infections in tsetse progeny was compromised, thus indicating its role in transmission to progeny [11]. Furthermore, previous studies have demonstrated that *Sodalis* motility genes, *fliC* and *motA*, and cell invasion genes, *invA1* and *invA2*, are up regulated in the larval and early pupal stages, and not in adult tsetse flies [12].

These studies led us to hypothesize that only at the larval stages *Sodalis* motility and invasion capabilities are activated and that these are essential factors to achieve an efficient colonization of the tsetse fly reproductive tissues and subsequent transmission to the offspring. This can be anticipated by introducing rec*Sodalis* during the new-born larval stage. Indeed, our data showed that rec*Sodalis* introduction via intralarval microinjection of third-instar larvae results in stably colonized adult tsetse flies. Moreover, midgut and female reproductive organs were efficiently colonized and rec*Sodalis* was stably transmitted throughout the subsequent progeny generations (evaluated until the 3rd generation in this study which corresponds to a total time period of around 150 days after the F_0_ larval injection). These results generate new questions regarding the biology of the factors that promote *Sodalis* colonization of tissues in the developing larva and their vertical transmission demanding further study.

## Conclusions

Collectively, this study demonstrates that rec*Sodalis* introduced via larval injection can be used to drive expression of exogenous transgenes in *G. m. morsitans* and that the recombinant bacteria can be transmitted to subsequent generations, similar to wild-type bacteria. This implies that a tsetse fly colony can be established with flies harboring a modified *Sodalis* of interest. This is a crucial step forward as it is an essential requirement for the mass rearing of paratransgenic tsetse flies as part of the SIT technique to control African trypanosomiasis in the field.

## Methods

### Insects, bacterial strains and culture conditions

*Glossina morsitans morsitans* (Westwood) from the colony at the Institute of Tropical Medicine Antwerp (ITM), that originated from pupae collected in Kariba (Zimbabwe) and Handeni (Tanzania), were used in all experiments. Flies, maintained at 26 °C and 65% relative humidity, were fed 3 days per week with defibrinated bovine blood (Slovak Academy of Sciences (SAS), Bratislava) using an artificial membrane system. *Sodalis glossinidius* strains used in this study were isolated from the hemolymph of surface-sterilized flies from the ITM colony. The generation of the GFP-tagged *Sodalis* strain has already been described [8] and was based on Tn7-mediated site-specific transposition. Briefly, plasmid pGRG25 [13] featuring the tnsABCD genes expressed under control of the pBAD promoter and a multiple cloning site, flanked by the left and right ends of Tn7 was used for transgene insertion into the chromosome of *S. glossinidius*. The *gfp* gene in fusion with either the *lacZ* or *GroEl* promotor was cloned into NotI-XhoI of the pGRG25 multiple cloning site, located between the Tn7R and Tn7L end sequences, at which the transposition proteins act to move the element. The plasmids were transformed into WT *S. glossinidius* cells using a heat shock method. Transformed cells were allowed to recover overnight at 26 °C prior to plating onto Mitsuhashi-Maramorosch (MM; PromoCell) -blood agar, supplemented with ampicillin. A single recombinant *S. glossinidius* colony was then inoculated into liquid culture and grown in the presence of arabinose allowing TnsABCD to be expressed leading to transgene insertion. Transposition was verified by PCR using primers that flank the *att*Tn7 site (GlmS_Fw: 5′ –TATGAAGATTATTCCCCTGCCGCA-3′; PhoS_rev: 5′- CCATTTAGCGTAAACCGGCG-3′) and sequencing of the PCR product. The pGRG25 plasmid was cured from recombinant *Sodalis* by multiple passages in the absence of ampicillin. Cultures were maintained in vitro at 26 °C in liquid MM insect medium supplemented with 10% (*v*/v) heat-inactivated fetal bovine serum (FBS).

### FACS analysis

The fluorescence intensity of the different chromosomally GFP-tagged *Sodalis* strains was measured using a FACSVerse flow cytometer (laser 488 nm (filter 527/32 with 507 LP filter)) (BD Biosciences) and data analysed using the BD FACSuite software package (version 1.0.5.3841). i) In vitro conditions; cells were washed once and resuspended in cold PBS at a final concentration of 5 × 10^6^ cells per ml. ii) In vivo; male teneral flies were briefly anaesthetized by cold shock and microinjected intrathoracically with a suspension of 5 × 10^6^ recombinant and WT *Sodalis* CFU. Injections were performed under a binocular microscope, using a 5 μl Hamilton 75RN microsyringe with gauge 30 removable electrotapered needles. After 7 days, hemolymph was collected from injected flies by removing one front fly leg at the joint nearest to the thorax and applying gentle pressure to the distal tip of the abdomen; 2 μl of hemolymph exuding from the wound was collected in 500 μl PBS using a 10 μl pipette..

### Recombinant symbiont introduction

#### Introduction in adult stages

Prior to rec*Sodalis* introduction, the (native) WT *Sodalis* population in adult experimental flies was suppressed via three subsequent blood meals supplemented with 20 μg/ml streptozotocin (Sigma). Three days after the last streptozotocin supplemented blood meal, rec*Sodalis* was introduced in these flies by 2 alternative routes: i) **Adult fly injection:** flies were briefly anaesthetized by cold shock (10 min at 4.0 °C) and injected intrathoracically using a 5 μl Hamilton 75RN 34 gauge microsyringe (with removable electro-tapered needles) with 5 × 10^6^ CFU rec*Sodalis* (CFU = colony forming units). ii) **Diet**: flies were fed a single rec*Sodalis* supplemented blood meal. Given that a single fly ingests approximately 25 μl of blood during one blood meal, blood was mixed to a concentration of 6.2 × 10^7^ CFU/ml rec*Sodalis*. Subsequently, approximately 1.5 × 10^6^ CFU rec*Sodalis* were ingested by a single fly. The mammalian complement system was inactivated by heating blood for 1 h at 56 °C.

#### Introduction in larval stages

Third-instar larvae, collected immediately after larviposition, were microinjected with 10^6^ CFU of *rec*Sodalis using a 5 μl Hamilton 75RN microsyringe with gauge 34 removable electrotapered needles and allowed to pupate. Injected pupae were maintained at 26 °C and 65% relative humidity until emergence 30 days later.

### qPCR based measuring of in vivo WT Sodalis and recSodalis densities

We used a quantitative PCR (qPCR) method for the estimation of the number of WT *Sodalis* and rec*Sodalis* cells in tsetse fly tissues (abdomen, thorax and reproductive tissue) [7]. Briefly, triplicate cultures of WT *Sodalis* and rec*Sodalis* were serially diluted (10-fold) in PBS to yield a *Sodalis* density ranging from 10^8^ CFU/ml to 10^2^ CFU/ml. DNA was extracted from each *Sodalis* dilution followed by qPCR using primers that amplify a region of the GFP gene present in rec*Sodalis*: (GFP_Fw, 5′- TGGCCAACACTTGTCACTAC-3′ and GFP_Rev, 5′- AGAAGGACCATGTGGTC -3′) and primers that target a 120-bp region of the single-copy *Sodalis glossinidius exochitinase* (Qchi) gene present in both WT and rec*Sodalis* to determine the corresponding Ct values. Standard curves were generated by plotting these C_t_ values against the corresponding log of *Sodalis* CFU/ml. This qPCR approach in combination with the *Sodalis* CFU standard curves allowed us to estimate the CFU (DNA equivalent) values present in the tsetse fly tissues of the different experimental fly series. For this, at various time points post-injection and post-larviposition, flies were sacrificed for genomic DNA extraction using the QIAGEN DNeasy extraction kit (QIAGEN). Dissections of abdomen, thorax, midgut and female reproductive organs were performed under a binocular microscope (Heerbrugg Switzerland), after sedation of the flies by cold shock (10 min at 4.0 °C).

An internal control was included to evaluate the DNA extraction efficiency in all the tissue samples. For this, samples were spiked with 0.32 ng of plasmid DNA (pCM66) prior to extraction. qPCR with pCM66*-*specific primers (pCM66_Fw: 5′- CTTGGCCCTCACTGACAG-3′ and pCM66__Rev, 5′- GCAGCCCTGGTTAAAAAC-3′) was performed on a selection of samples to control the extraction efficiency.

qPCR was performed in a 20-μl reaction mixture volume containing 10 μl of 2 × iQ™ SYBR green supermix, 0.3 μM of each primer, template (genomic DNA, standard) and RNase-free sterile water to a final volume of 20 μl. The amount of fluorescence generated was measured during each amplification cycle using the following program: (i) initial denaturing at 95 °C for 10 min; (ii) 40 cycles, with 1 cycle consisting of denaturation at 95 °C for 10 s, annealing at 60 °C for 10 s, and extension at 72 °C for 30 s. All assays were carried out on a LightCycler™ (Roche Diagnostics, Mannheim, Germany) in 96-well format plates in duplicate and were averaged for each sample. For each PCR run, a negative (no-template) control was used to test for false-positive results or contamination.

## Additional file

Additional file 1: **Figure S1.** Number of WT *Sodalis* CFU in abdomen, thorax and reproductive tissues of streptozotocin-treated (red circle) and non-treated (black ■) female flies. Prior to injection, treated flies were given three blood meals supplemented with 20 μg/ml streptozotocin during the first week after emergence, while non-treated flies received normal blood meals. Data points represent the mean *Sodalis* CFU (± SD) present in the different tissues of at least 5 individual flies at the time of sampling; 4 days post-eclosion. The number of CFU is represented in log scale on the y-axis. (DOCX 19 kb)

## Abbreviations

CFU: Colony forming unit
GFP: Green fluorescent protein
Rec*Sodalis*: GFP-tagged *Sodalis*
SIT: Sterile insect technique
WT: Wild type
