# Supplementary material for: Towards improving tsetse fly paratransgenesis: stable colonization of Glossina morsitans morsitans with genetically modified Sodalis
Source: BMC Microbiol. 2018 Nov 23;18(Suppl 1):165. doi: 10.1186/s12866-018-1282-9 (PMC6251102; doi:10.1186/s12866-018-1282-9)
Supplement: Supplementary file 1 — Figure S1. Number of WT Sodalis CFU in abdomen, thorax and reproductive tissues of streptozotocin-treated (red circle) and non-treated (black ■) female flies. Prior to injection, treated flies were given three blood meals supplemented with 20 μg/ml streptozotocin during the first week after emergence, while non-treated flies received normal blood meals. Data points represent the mean Sodalis CFU (± SD) present in the different tissues of at least 5 individual flies at the time of sampling; 4 days post-eclosion. The number of CFU is represented in log scale on the y-axis. (DOCX 19 kb) [file 12866_2018_1282_MOESM1_ESM.docx]

**Additional file 1**

**Figure S1 - Number of WT *Sodalis* CFU in abdomen, thorax and reproductive tissues of streptozotocin-treated (red ●) and non-treated (black ■) female flies.**

Prior to injection, treated flies were given three blood meals supplemented with 20 μg/ml streptozotocin during the first week after emergence, while non-treated flies received normal blood meals. Data points represent the mean *Sodalis* CFU (± SD) present in the different tissues of at least 5 individual flies at the time of sampling; 4 days post-eclosion. The number of CFU is represented in log scale on the y-axis.
